# On Thermodynamical Kluitenberg Theory in General Relativity

**DOI:** 10.3390/e27080833

**Published:** 2025-08-06

**Authors:** Francesco Farsaci, Patrizia Rogolino

**Affiliations:** Department of Mathematical and Computer Sciences, Physical Sciences and Earth Sciences, University of Messina, Viale F. Stagno d’Alcontres, 31, 98166 Messina, Italy; farsaci@ipcf.cnr.it

**Keywords:** non-equilibrium thermodynamics, kluitenberg general relativity, entropy production, four-dimensional thermodynamical representation

## Abstract

In this paper, we introduce Kluitenberg’s formulation of non-equilibrium thermodynamics with internal variables in the context of a Riemannian space, as required by Einstein’s general relativity. Using the formulation of the second law of thermodynamics in general coordinates with a pseudo-Euclidean metric, we derive a Levi-Civita-like energy tensor and propose a generalization of the second law within a Riemannian space, in agreement with Tolman’s approach. In addition, we determine the expression for the entropy density in a general Riemannian space and identify the new variables upon which it depends. This allows us to deduce, within this framework, the equilibrium inelastic and viscous stress tensors as well as the entropy production. These expressions are consistent with the principle of general covariance and Einstein’s equivalence principle.

## 1. Introduction

Modern theoretical methods of non-equilibrium thermodynamics (NET), through a suitable choice of variables, allow one to summarize and, to some extent, classify the behavior of materials using a limited set of parameters known as phenomenological and state coefficients [1]. These coefficients, fundamental in NET, relate entropy sources to irreversible processes occurring within a medium, thus describing essential physical phenomena taking place in the system under study. A central aspect of this formalism is the definition of thermodynamic generalized forces (also known as affinities) and fluxes, which together account for entropy variation. These quantities are connected by phenomenological equations which, in many cases, are assumed to be linear. The coefficients appearing in such linear relations, called phenomenological coefficients [1], are often treated as constant in time, although they actually vary dynamically and depend on the frequency spectrum of the excitations. Recognizing that standard thermodynamic variables are sometimes insufficient to describe certain phenomena, Kluitenberg introduced the concept of internal variables, represented by vector fields acting as thermodynamic internal degrees of freedom [2,3]. These variables account for various relaxation phenomena, such as mechanical or dielectric relaxations [4,5]. This theoretical framework enables the formulation of additional vector fields, dependent on the phenomenological and state coefficients, that provide a more complete description of relaxation behavior in materials.

In our previous paper [6,7], Kluitenberg’s non-equilibrium thermodynamics was extended to include variables within special relativity. This formulation is based on the principle of relativity, which expresses the invariability of the laws of physics under a change between two inertial reference frames. In [6], after determining the transformation law for the strain tensor, we assumed that the entropy density in an arbitrary inertial reference frame depends on the energy density (expressed by means of the temporal component of the energy tensor), the relativistic strain tensor, and the anelastic part of the total strain tensor. This allowed us to determine the law of temperature transformation, namely Ott’s law [8], and to introduce the equilibrium and affinity stress tensor in a general inertial frame. Through these entities, it has been possible to determine the relativistic entropy production. Furthermore, the transformation law of the elastic tensor [9] and the relativistic phenomenological equations, along with the transformation law of the corresponding coefficients, were derived. In [6], these results have been obtained in three–dimensional form.

Following this framework, the present paper aims to develop a consistent formulation of thermodynamic quantities, such as the viscous stress tensor and the equilibrium stress tensor, within a relativistic framework. These quantities can be regarded as terms of the energy-momentum tensor. The derivation of their transformation laws under general coordinate changes is essential to ensure the consistency of any relativistic theory, particularly when dealing with non-inertial frames or curved spacetimes. Furthermore, we emphasize that, unlike in Newtonian mechanics, where the metric tensor $g_{\mu \nu }$ is Euclidean, in our treatment, the metric tensor is not Euclidean. Instead, it respects the equivalence principle and employs a covariant formulation suitable for a general relativistic setting. In particular, in the present paper, we focus on mechanical phenomena and attempt a further generalization of the aforementioned theory to general relativity. Since this requires a four-dimensional approach, we begin by recalling some concepts from special relativistic thermodynamics, which provide the foundation for the four-dimensional formulation. Our approach is in agreement with Tolman’s framework [10,11,12].

The paper is organized as follows. In Section 2, we briefly recall the second law in special relativity and illustrate the four-dimensional formalism. In Section 3, we introduce Riemannian space and derive the energy tensor. The generalization of the Kluitenberg’ approach to general relativity is developed in Section 4. In Section 5, the balance equation for entropy and the corresponding entropy production are derived. Finally, in Section 6, we provide concluding remarks on the proposed theory.

## 2. Remarks on the Second Law of Thermodynamics in Special Relativity: Four-Dimensional Form

Let *A* be an elementary portion of a continuous medium with a volume δv, and let ϕ be the entropy density evaluated at the point *P* of *A*. The entropy of the element *A* will be ϕδv and the second law of thermodynamics can be written as follows [10]:

(1) $$\frac{d}{dt}\left(\phi \delta v\right)\delta t\geq \frac{\delta Q}{T}.$$

This relation connects the entropy variation of *A* during the time interval δt to the heat δQ that flows inside *A* supplied at the temperature *T* in time δt. From relation (1), it follows that

(2) $$\left[\frac{d\phi }{dt}\delta v+\phi \frac{d(\delta v)}{dt}\right]\delta t\geq \frac{\delta Q}{T}.$$

Recalling that

$$\frac{d(\delta v)}{dt}=divu\;\delta v,$$

where u is the fluid velocity, Equation (2) becomes

(3) $$\left(\frac{d\phi }{dt}\delta v+\phi \frac{du_{i}}{dx_{i}}\delta v\right)\delta t\geq \frac{\delta Q}{T}.$$

Taking into account that (see eq. 4.3 of [6])

(4) $$\phi =\phi (x_{i}\;|t),\;i=1,2,3$$

after using the Einstein summation convention, it follows that

(5) $$\begin{array}{ccc} & & \left[\left(\frac{\partial \phi }{dx_{i}}\frac{dx_{i}}{dt}+\frac{\partial \phi }{\partial t}\right)\delta v+\phi \frac{\partial u_{i}}{\partial x_{i}}\delta v\right]\delta t\geq \frac{\delta Q}{T}, \end{array}$$

(6) $$\begin{array}{ccc} & & \left(\frac{\partial (\phi \;u_{i})}{\partial x_{i}}+\frac{\partial \phi }{\partial t}\right)\delta v\;\delta t\geq \frac{\delta Q}{T}. \end{array}$$

We state that the Latin index assumes the values 1,2,3 while the Greek index takes the values 0,1,2,3. Let us consider the well-known pseudo-Euclidean metric [13,14,15]

(7) $$ds^{2}={\left(dx^{0}\right)}^{2}-{\left(dx^{i}\right)}^{2},$$

wherein $x^{0}=ct$ and *c* is the speed of light in a vacuum. In this space, the relation (4) can be rewritten as

(8) $$\phi =\phi (x^{\mu }),\;\mu =0,1,2,3$$

and the expression (6) becomes

(9) $$\left(\frac{\partial }{\partial x^{i}}\left(\phi \frac{dx^{i}}{dx^{0}}\right)+\frac{\partial \phi }{\partial x^{0}}\right)\delta x^{0}\;\delta x^{1}\;\delta x^{2}\;\delta x^{3}\geq \frac{\delta Q}{T}.$$

Dividing Equation (7) by ${\left(dx^{0}\right)}^{2}$, we obtain

(10) $$\frac{ds}{dx^{0}}=\sqrt{1-\frac{V^{2}}{c^{2}}}.$$

Moreover, the following relation is well known [6,10]

(11) $$\phi =\frac{\phi_{0}}{\sqrt{1-\frac{V^{2}}{c^{2}}}}$$

or

(12) $$\phi_{0}=\phi \sqrt{1-\frac{V^{2}}{c^{2}}},$$

where $\phi_{0}$ is the entropy density in the proper frame. Taking into account Equations (10) and (12), the inequality (9) becomes

(13) $$\left[\frac{\partial }{\partial x^{\mu }}\left(\phi_{0}\frac{dx^{\mu }}{ds}\right)\right]\delta x^{0}\;\delta x^{1}\;\delta x^{2}\;\delta x^{3}\geq \frac{\delta Q}{T}.$$

By defining the entropy density four-vector $S^{\mu }$ as follows [11,12],

(14) $$S^{\mu }=\phi_{0}\frac{dx^{\mu }}{ds},$$

Equation (13) assumes the following form [10,16,17]

(15) $$\frac{\partial S^{\mu }}{\partial x^{\mu }}\;\delta x^{0}\;\delta x^{1}\;\delta x^{2}\;\delta x^{3}\geq \frac{\delta Q_{0}}{T_{0}},$$

where the following well-known equation is used:

(16) $$\frac{\delta Q}{T}=\frac{\delta Q_{0}}{T_{0}},$$

from Tolman (1958) [10], as this expression remains valid due to the invariance of the ratio of heat to temperature under Lorentz transformations. In relation (16), the terms $\delta Q_{0}$ and $T_{0}$ represent the heat flux and the temperature in the proper system, respectively. This equation is introduced within the framework of special relativity. As for the definition of temperature in general relativity, it has been defined, following the ideas of Levi-Civita, as a natural generalization of the special relativistic case, in accordance with the definition proposed in one of our previous papers, and consistent with Ott’s definition. In any case, the formulation respects the equivalence principle.

## 3. Riemannian Space and Energy Tensor

It is evident that the above Equations (8)–(16) refer to the metric (7), and the derivatives that appear in these equations are not of the tensor type. Now we attempt to extend Equation (15) to general relativity. For this purpose, we first introduce the energy tensor following Levi-Civita’s meaning for the components. If

(17) $$ds^{2}=g_{\mu \nu }dx^{\mu }dx^{\nu }$$

is the metric of Riemannian space, Levi-Civita states (here, and only here, the Einstein summation convention does not apply) [18]:

$$\frac{T_{ik}}{\sqrt{g_{ii}\;g_{kk}}}=\frac{T_{ki}}{\sqrt{g_{ii}\;g_{kk}}},$$

which represents the component (normal) along the $x^{i}$ direction (where only $x^{i}$ changes) of the stress acting on a surface element normal to the $x^{k}$ direction (or vice versa swapping *i* with *k*). The quantity

$$\frac{T_{i0}}{\sqrt{-g_{00}\;g_{ii}}}=\frac{T_{0i}}{\sqrt{-g_{00}\;g_{ii}}}$$

is the component along the $x^{i}$ direction of energy flow in a light-second, and the component

$$\frac{T_{00}}{g_{00}}$$

represents the distribution of energy density in the space $x^{1},x^{2},x^{3}$ [18].

Furthermore, Equation (17) can be written as

(18) $$ds^{2}=g_{00}{\left(dx^{0}\right)}^{2}+2g_{0i}dx^{0}dx^{i}+g_{ik}dx^{i}dx^{k},$$

where we recall that $x^{0}=ct$ with *c* being the scalar speed of light in vacuum, which is considered constant, while the speed of light in the presence of gravitation is given by $c\sqrt{g_{00}}$.

If we define

(19) $$dl^{2}=-g_{ik}dx^{i}dx^{k},$$

which represents the infinitesimal distance between two points on the spatial line *l* [13]; Equation (18) becomes

(20) $$ds^{2}=g_{00}{\left(dx^{0}\right)}^{2}+2g_{0i}dx^{0}dx^{i}-dl^{2},$$

from which it follows that

(21) $$\frac{ds}{dx^{0}}=\sqrt{g_{00}+2g_{0i}\frac{dx^{i}}{dx^{0}}-\frac{v^{2}}{c^{2}}},$$

where $v=\frac{dl}{dt}$ is the velocity of the particle. Moreover,

$$\frac{dx^{i}}{dx^{0}}=\frac{1}{c}\frac{dx^{i}}{dl},\frac{dl}{dt}=\frac{dx^{i}}{dl}\frac{v}{c}$$

and Equation (21) becomes

(22) $$\frac{ds}{dx^{0}}=\sqrt{g_{00}+2g_{0i}\frac{dx^{i}}{dl}\frac{v}{c}-\frac{v^{2}}{c^{2}}},$$

from which it follows that

(23) $$\frac{dx^{0}}{ds}=\frac{1}{\sqrt{g_{00}+2g_{0i}\frac{dx^{i}}{dl}\frac{v}{c}-\frac{v^{2}}{c^{2}}}}.$$

Equation (22) satisfies the principle of equivalence because, if the metric (20) is identified with relation (7), then Equation (22) reduces to Equation (10) [19,20].

## 4. Generalization to General Relativity

The natural generalization of Equation (15), which satisfies both the equivalence principle and general covariance, is given by [10,11,12,21,22]

(24) $$G_{/\mu }^{\;\mu }\delta \Gamma \geq \frac{\delta Q_{0}}{T_{0}},$$

where

(25) $$G^{\mu }=S^{\mu }\sqrt{-g},$$

$g=\left|\right|g_{\mu \nu }\left|\right|$ and

(26) $$\delta \Gamma =dx^{0}dx^{1}dx^{2}dx^{3}.$$

The quantity $S^{\mu }$ that appears in Equation (25) is evaluated taking into account the metric given by Equation (20). It can be shown that Equation (24) can be written [11,12]:

(27) $$S_{/\mu }^{\mu }\sqrt{-g}\;\delta \Gamma ={\left(\phi_{0}\frac{dx^{\mu }}{dx^{0}}\right)}_{/\mu }\sqrt{-g}\delta \Gamma =\frac{\partial }{\partial x^{\mu }}\left(\phi_{0}\frac{dx^{\mu }}{ds}\sqrt{-g}\right)\delta \Gamma \geq \frac{\delta Q_{0}}{T_{0}}$$

or

(28) $$\frac{\partial }{\partial x^{\mu }}\left(\phi_{0}\frac{dx^{\mu }}{ds}\sqrt{-g}\right)\delta \Gamma \geq \frac{\delta Q_{0}}{T_{0}}.$$

Here, we do not explain the physical content of Equations (24)–(28), for further detail, and the reader may refer to Tolman’s exposition [10]. Furthermore, for our purpose, we consider the four-vector given in Equation (23):

(29) $$G^{\mu }=\phi_{0}\frac{dx^{\mu }}{ds}\sqrt{-g}.$$

In particular, we focus on its time component

(30) $$G^{0}=\phi_{0}\frac{dx^{0}}{ds}\sqrt{-g}.$$

After substituting Equation (23) we obtain

(31) $$G^{0}=\frac{\phi_{0}}{\sqrt{g_{00}+2g_{0i}\frac{dx^{i}}{dl}\frac{v}{c}-\frac{v^{2}}{c^{2}}}}\sqrt{-g}.$$

This equation reduces to Equation (11) when the metric (20) becomes the one expressed by (7) [6]. Now, observing that Equation (11) represents the expression of the entropy density in a generic inertial frame, it is reasonable to interpret Equation (31) as its extension to a Riemannian space with metric (20). Therefore, we consider $G^{0}$, as defined in Equation (31), to represent the entropy density in such a space. By analogy with Equation (11), we define

(32) $$G^{0}=\phi_{g},$$

where the subscript *g* stands for “general”. Rewriting Equation (31) in this form, we obtain

(33) $$\phi_{g}=\frac{\phi_{0}}{\sqrt{g_{00}+2g_{0i}\frac{dx^{i}}{dl}\frac{v}{c}-\frac{v^{2}}{c^{2}}}}\sqrt{-g}.$$

This expression also satisfies the equivalence principle [20]. In the following, a bar over a quantity indicates that it is evaluated in the proper frame. At this point, a natural question arises: what are the variables upon which entropy density (33) depends, in a frame of reference with the metric given by (20)?

As previously mentioned, $\phi_{0}$ depends on the energy density (given by the temporal component of the energy tensor ${\bar{T}}^{00}$), as well as on the total strain tensor ${\bar{\gamma }}^{ik}$ and the anelastic part of the total strain tensor ${\bar{\gamma }}_{\left(1\right)}^{ik}$ according to [6]:

(34) $$\phi_{0}=\phi_{0}\left({\bar{T}}^{00}|{\bar{\gamma }}^{ik},{\bar{\gamma }}_{\left(1\right)}^{ik}\right).$$

In special relativity, this dependence is preserved by invoking the relativity principle [6]. In that context, the entropy density expressed by Equation (11) was considered a function of the transformed quantities ${\bar{T}}^{00},{\bar{\gamma }}^{ik},{\bar{\gamma }}_{\left(1\right)}^{ik}$ as derived in [6]. The considerations made in [6] were based exclusively on the principle of relativity and, consequently, on Lorentz transformations. However, since we are now extending the discussion to general relativity, it becomes necessary to consider general coordinate transformations [13,16]:

(35) $$x^{\alpha }=x^{\alpha }\left({\bar{x}}^{\beta }\right),$$

which admits the inverse transformation

(36) $${\bar{x}}^{\beta }={\bar{x}}^{\beta }\left(x^{\alpha }\right).$$

Unlike in special relativity, we cannot postulate a principle of general relativity, as such a principle is not physically meaningful within the context of general relativity. Consequently, generalizing the form of Equation (34) is non-trivial in this new framework.

The only two principles we have to satisfy are [10]

**(a)**  The general covariance principle
**(b)**  The equivalence principle.

However, the following considerations allow us to obtain a functional dependence similar to that exepressed by Equation (34), but with a different meaning of the variables. Obviously, the functions ${\bar{T}}^{00},{\bar{\gamma }}^{ik},{\bar{\gamma }}_{\left(1\right)}^{ik}$, on which depends $\phi_{0}$, will have the corresponding form in a generic reference frame, and their law of transformation will follow those of tensors under a general transformation of coordinates, as given by Equation (35). Therefore, for the component ${\bar{T}}^{00}$ we have [13]

(37) $${\bar{T}}^{00}=\frac{\partial {\bar{x}}^{0}}{\partial x^{\mu }}\frac{\partial {\bar{x}}^{0}}{\partial x^{\nu }}T^{\mu \nu },$$

where $T^{\mu \nu }$ is the energy-momentum tensor in the general frame. Equation (37) shows that

(38) $${\bar{T}}^{00}={\bar{T}}^{00}\left(T^{\alpha \beta }\right).$$

The term ${\bar{T}}^{00}$ in this approach depends on the tensor $T^{\alpha \beta }$, and not just on $T^{00}$ as in special relativity [6]. All this, taking into account Equation (33), allows us to consider $\phi_{g}$ as function of $T^{\alpha \beta }$.

As for the correspondence of ${\bar{\gamma }}_{\left(0\right)}^{ik}$ and ${\bar{\gamma }}_{\left(1\right)}^{ik}$ in a generic frame, denoted by $\gamma_{\left(0\right)}^{ik}$ and $\gamma_{\left(1\right)}^{ik}$ respectively, it is important to observe that, under a general coordinate transformation, the physical meaning of the phenomena described by ${\bar{\gamma }}_{\left(0\right)}^{ik}$ and ${\bar{\gamma }}_{\left(1\right)}^{ik}$ may change.

In other words, due to the presence of a gravitational field, an elastic phenomenon can, for example, be transformed into an inelastic one. Therefore, under a general coordinate transformation (35), we never have a purely elastic phenomenon.

Likewise, an inelastic phenomenon can be transformed into an elastic one. Because ${\bar{\gamma }}_{\left(0\right)}^{ik}$ and ${\bar{\gamma }}_{\left(1\right)}^{ik}$ correspond to $\gamma_{\left(0\right)}^{ik}$ and $\gamma_{\left(1\right)}^{ik}$, respectively, according to the transformation law of the tensors, if [2,3,6]

(39) $${\bar{\gamma }}^{ik}={\bar{\gamma }}_{\left(0\right)}^{ik}+{\bar{\gamma }}_{\left(1\right)}^{ik}$$

then we can naturally define the corresponding of ${\bar{\gamma }}^{ik}$ as

(40) $$\gamma^{ik}=\gamma_{\left(0\right)}^{ik}+\gamma_{\left(1\right)}^{ik}.$$

The transformation law of tensors allows us to write the following functional dependences:

(41) $$\begin{array}{ccc} & & \gamma_{\left(0\right)}^{ik}=\gamma_{\left(0\right)}^{ik}\left({\bar{\gamma }}_{\left(0\right)}^{rs}\right) \end{array}$$

(42) $$\begin{array}{ccc} & & \gamma_{\left(1\right)}^{ik}=\gamma_{\left(1\right)}^{ik}\left({\bar{\gamma }}_{\left(1\right)}^{rs}\right) \end{array}$$

remembering the differences in the physical meaning of the phenomena to which the strain tensor refers in the proper system and in a generic system. These considerations allow us to write the following relation:

(43) $$\phi_{g}=\phi_{g}\left(T^{00}|\gamma^{ik},\gamma_{\left(1\right)}^{ik}\right).$$

From Equations (28), (38) and (43), it follows that

(44) $$\frac{\partial \phi_{g}}{\partial T^{\alpha \beta }}=\frac{\partial \phi_{0}}{\partial {\bar{T}}^{00}}\frac{\partial {\bar{T}}^{00}}{\partial T^{\alpha \beta }}\frac{dx^{0}}{ds}\sqrt{-g},$$

whose components are

(45) $$\begin{array}{ccc} & & \frac{\partial \phi_{g}}{\partial T^{00}}=\frac{\partial \phi_{0}}{\partial {\bar{T}}^{00}}\frac{\partial {\bar{T}}^{00}}{\partial T^{00}}\frac{dx^{0}}{ds}\sqrt{-g} \end{array}$$

(46) $$\begin{array}{ccc} & & \frac{\partial \phi_{g}}{\partial T^{i0}}=\frac{\partial \phi_{g}}{\partial T^{0i}}=\frac{\partial \phi_{0}}{\partial {\bar{T}}^{00}}\frac{\partial {\bar{T}}^{00}}{\partial T^{0i}}\frac{dx^{0}}{ds}\sqrt{-g} \end{array}$$

(47) $$\begin{array}{ccc} & & \frac{\partial \phi_{g}}{\partial T^{ik}}=\frac{\partial \phi_{g}}{\partial T^{ki}}=\frac{\partial \phi_{0}}{\partial {\bar{T}}^{00}}\frac{\partial {\bar{T}}^{00}}{\partial T^{ik}}\frac{dx^{0}}{ds}\sqrt{-g} \end{array}$$

For analogy with special relativity, we define the temperature *T* in a general frame of reference [6]

(48) $$\frac{1}{T}\frac{\partial \phi_{g}}{\partial T^{00}}=\frac{1}{\bar{T}}\frac{\partial {\bar{T}}^{00}}{T^{00}}\frac{dx^{0}}{ds}\sqrt{-g},$$

where

(49) $$\frac{1}{\bar{T}}=\frac{\partial \phi_{0}}{\partial T^{00}}$$

is the temperature in the proper frame [6]. By defining

(50) $$\frac{1}{\chi }=\frac{\partial {\bar{T}}^{00}}{\partial T^{00}}\frac{dx^{0}}{ds}\sqrt{-g},$$

Equation (48) becomes

(51) $$T=\chi \bar{T}.$$

This equation satisfies the equivalence principle because Equation (50) becomes

(52) $$\chi =\sqrt{1-\frac{V^{2}}{c^{2}}}=\alpha .$$

If the metric is defined by relation (7) [6], then Equation (52) can be written as follows

(53) $$T=\alpha \bar{T},$$

in accordance with the Ott transformation law of special relativity [8]. It can be observed that Equation (46) represents the entropy variation due to variation of the components of the energy flow along the $x^{i}$ direction, while Equation (47) represents the entropy variation due to components of the stress exerted on a surface element normal to the $x^{k}$ direction. From Equations (33), (34), and (43), it follows that

(54) $$\frac{\partial \phi_{g}}{\partial \gamma^{ik}}=\frac{\partial \phi_{0}}{\partial {\bar{\gamma }}^{rs}}\frac{\partial {\bar{\gamma }}^{rs}}{\partial \gamma^{ik}}\frac{dx^{0}}{ds}\sqrt{-g}.$$

It is well known that [6]

(55) $$\frac{\partial \phi_{0}}{\partial {\bar{\gamma }}^{rs}}=-\frac{1}{\bar{T}}{\bar{\tau }}_{rs}^{\left(eq\right)},$$

where ${\bar{\tau }}_{rs}^{\left(eq\right)}$ is the equilibrium stress tensor in the proper system [2,3,6]. Then, Equation (54) becomes

(56) $$\frac{\partial \phi_{g}}{\partial \gamma^{ik}}=-\frac{1}{\bar{T}}\frac{\partial {\bar{\gamma }}^{rs}}{\partial \gamma^{ik}}\frac{dx^{0}}{ds}\sqrt{-g}\;{\bar{\tau }}_{rs}^{\left(eq\right)}.$$

From Equation (48), one has

(57) $$\frac{1}{\bar{T}}\frac{dx^{0}}{ds}\sqrt{-g}=\frac{1}{T\;\frac{\partial {\bar{T}}^{00}}{\partial T^{00}}}$$

and Equation (56) can be written as

(58) $$\frac{\partial \phi_{g}}{\partial \gamma^{ik}}=-\frac{1}{T}\;{\bar{\tau }}_{rs}^{\left(eq\right)}\frac{\frac{\partial {\bar{\gamma }}^{rs}}{\partial \gamma^{ik}}}{\frac{\partial {\bar{T}}^{00}}{\partial T^{00}}}$$

or

(59) $$T\frac{\partial \phi_{g}}{\partial \gamma^{ik}}=-{\bar{\tau }}_{rs}^{\left(eq\right)}\frac{\frac{\partial {\bar{\gamma }}^{rs}}{\partial \gamma^{ik}}}{\frac{\partial {\bar{T}}^{00}}{\partial T^{00}}}.$$

Now, we define the equilibrium stress tensor $\tau_{rs}^{\left(eq\right)}$ in a generic frame, with the metric given by Equation (20), as

(60) $$\tau_{rs}^{\left(eq\right)}=-T\frac{\partial \phi_{g}}{\partial \gamma^{rs}},$$

by analogy with special relativity [6]. Then, Equation (59) becomes

(61) $$\tau_{ik}^{\left(eq\right)}=\frac{{\bar{\tau }}_{rs}^{\left(eq\right)}}{\frac{\partial {\bar{T}}^{00}}{\partial T^{00}}}\frac{\partial {\bar{\gamma }}^{rs}}{\partial \gamma^{ik}}.$$

Equations (59)–(61) satisfy the equivalence principle if the metric is expressed by relation (7) (see [6]). From Equation (43), following a similar procedure, we define the affine stress tensor $\tau_{ik}^{\left(1\right)}$ as

(62) $$\tau_{ik}^{\left(1\right)}=\frac{{\bar{\tau }}_{rs}^{\left(1\right)}}{\frac{\partial {\bar{T}}^{00}}{\partial T^{00}}}\frac{\partial {\bar{\gamma }}^{rs}}{\partial {\gamma_{\left(1\right)}}^{ik}},$$

and

(63) $$\tau_{rs}^{\left(1\right)}=-T\frac{\phi_{g}}{\partial {\gamma^{rs}}_{\left(1\right)}}.$$

This also satisfies the equivalence principle, as can be easily shown. Equations (61) and (62) express a transformation law for stress tensors. Since this law must be the same for each type of stress, it follows that

(64) $$\frac{\frac{\partial {\bar{\gamma }}^{rs}}{\partial \gamma^{ik}}}{\frac{\partial {\bar{T}}^{00}}{\partial T^{00}}}=\frac{\frac{\partial {\bar{\gamma^{rs}}}_{\left(1\right)}}{\partial {\gamma^{ik}}_{\left(1\right)}}}{\frac{\partial {\bar{T}}^{00}}{\partial T^{00}}}$$

or

(65) $$\frac{\partial {\bar{\gamma }}^{rs}}{\partial \gamma^{ik}}=\frac{\partial {\bar{\gamma^{rs}}}_{\left(1\right)}}{\partial {\gamma^{ik}}_{\left(1\right)}}.$$

Here, we assume that even in general relativity, the gravitational field does not influence the meaning of stress, which is naturally defined as the ratio of force to surface. Therefore, the transformation of the generic stress tensor can be obtained from the following tensor:

(66) $$T_{ik}^{rs}=\frac{\frac{\partial {\bar{\gamma }}^{rs}}{\partial \gamma^{ik}}}{\frac{\bar{\partial }T^{00}}{\partial T^{00}}}.$$

This is a fourth-rank tensor with 81 components. Now it is easy to adopt a special relativity approach to thermodynamics and introduce the viscous stress tensor. Recalling that in a proper frame, the viscous stress tensor is defined as [2,3,6]:

(67) $${\bar{\tau }}_{ik}^{\left(vi\right)}={\bar{\tau }}_{ik}-{\bar{\tau }}_{ik}^{\left(eq\right)},$$

we define the viscous stress tensor $\tau_{rs}^{\left(vi\right)}$ in a general frame of reference with metric given by Equation (20), as the transformation of tensor (67) via tensor (66):

(68) $$\tau_{rs}^{\left(vi\right)}=T_{rs}^{ik}{\bar{\tau }}_{ik}^{\left(vi\right)}.$$

Here too, the principle of equivalence holds.

We define specific contributions to the energy–momentum tensor, namely those given by Equations (61), (62), (67) and (68), which nevertheless satisfy the equivalence principle. Naturally, these are not equations but rather tensorial expressions incorporated into the energy–momentum tensor appearing in Einstein’s field equations. We consider the energy–momentum tensor in various formulations, but for our purposes, we restrict attention to a subset of its contributions (see Equations (38) and (45)–(53)), assuming the metric given by Equation (17). We have considered the metric tensor, which is used to express the gravitational field tensor, and therefore, we are implicitly accounting for Einstein’s field equations.

## 5. Balance Equation of Entropy and Entropy Production

On the basis of the relations deduced so far, in this section, we derive the balance equation and the expression of the entropy production in the formalism of general relativity. From Equations (29), (31) and (33) it follows

(69) $$\frac{\partial G^{\mu }}{\partial x^{\mu }}=\frac{\partial \phi_{g}}{\partial x_{0}}+\frac{\partial G^{i}}{\partial x^{i}}.$$

Observing that

(70) $$\frac{dx^{i}}{ds}=\frac{v^{i}}{c}\frac{dx^{0}}{ds},$$

it follows from Equation (29)

(71) $$G^{i}=\frac{\phi_{0}}{\sqrt{g_{00}^{2}+2g_{0i}\frac{dx^{i}}{dl}\frac{v}{c}-\frac{v^{2}}{c^{2}}}}\frac{v^{i}}{c}\sqrt{-g},$$

and hence Equation (69) becomes

(72) $$\frac{\partial G^{\mu }}{\partial x^{\mu }}=\frac{\partial \phi_{g}}{\partial x^{0}}+\frac{1}{c}\frac{\partial (\phi_{g}v^{i})}{\partial x^{i}},$$

which expresses the balance equation of the entropy density. The term $\frac{\partial \phi_{g}}{\partial x^{0}}$ represents an entropy production (source), whereas the term $\frac{1}{c}\frac{\partial \phi_{(}gv^{i})}{\partial x^{i}}$ represents the divergence of an entropy flux. Here, we provide an explicit expression for the entropy production in which the stress and strain appear. Introducing

(73) $$\sigma =\frac{dx^{0}}{ds}\sqrt{-g}$$

and taking into account relations (34) and (43), Equation (33) becomes

(74) $$\phi_{g}\left(T^{\alpha \beta }|\gamma^{ik},\gamma_{\left(1\right)}^{ik}\right)=\sigma \phi_{0}\left({\bar{T}}^{00}|,{\bar{\gamma }}^{ik},{\bar{\gamma }}_{\left(i\right)}^{ik}\right),$$

from which it follows that

(75) $$\frac{\partial \phi_{g}}{\partial x^{0}}=\frac{\partial \phi_{g}}{\partial T^{\alpha \beta }}\frac{\partial T^{\alpha \beta }}{\partial x^{0}}+\frac{\partial \phi_{g}}{\partial \gamma^{ik}}\frac{\partial \gamma^{ik}}{\partial x^{0}}+\frac{\partial \phi_{g}}{\partial \gamma_{\left(1\right)}^{ik}}\frac{\partial \gamma_{\left(1\right)}^{ik}}{\partial x^{0}}.$$

Substituting Equation (44) into (75), and taking into account relation (49), we obtain

(76) $$\frac{\partial \phi_{g}}{\partial x^{0}}=\frac{1}{\partial \bar{T}}\frac{\partial {\bar{T}}^{00}}{\partial x^{0}}\sigma -\frac{1}{T}\tau_{ik}^{\left(eq\right)}\frac{\partial \gamma^{ik}}{\partial x^{0}}+\frac{1}{T}\tau_{ik}^{\left(1\right)}\frac{\partial \gamma_{\left(1\right)}^{ik}}{\partial x^{0}}.$$

Considering the transformations (35) and (36), Equation (76) can be rewritten as

(77) $$\frac{\partial \phi_{g}}{\partial x^{0}}=\frac{\sigma }{\bar{T}}\frac{\partial {\bar{T}}^{00}}{\partial {\bar{x}}^{\alpha }}\frac{\partial {\bar{x}}^{\alpha }}{\partial x^{0}}-\frac{1}{T}\tau_{ik}^{\left(eq\right)}\frac{\partial \gamma^{ik}}{\partial x^{0}}+\frac{1}{T}\tau_{ik}^{\left(1\right)}\frac{\partial \gamma_{\left(1\right)}^{ik}}{\partial x^{0}}.$$

This is the explicit form of entropy production in general relativity. It is easy to prove that it satisfies the principle of equivalence.

## 6. Conclusions

Non-equilibrium thermodynamics in general relativity finds significant application in cosmology, particularly in analyzing the large-scale behavior of cosmic fluids, as well as in studying galaxy formation and the evolution of mass and energy densities through the energy–momentum tensor. As such, it proves to be especially suited to investigating the expansion of the universe. A more detailed approach, based on non-equilibrium thermodynamics with internal variables, can therefore make a substantial contribution to the understanding of these phenomena. In this framework, the energy–momentum tensor plays a central role, and a refined formulation of its contributions becomes necessary. This paper aims precisely to introduce specific constituents of the energy-momentum tensor, including various types of stress tensors, within a general relativistic framework that incorporates internal variables. Such a tensor is, of course, an integral part of Einstein’s field equations.

The extension of Kluitenberg’s non-equilibrium thermodynamics with internal variables to general relativity has been developed using the Levi-Civita formalism to construct the energy tensor. Additionally, the Tolman approach was adopted to formulate special relativity in four-dimensional pseudo-Euclidean space, and its extension to Riemannian space was achieved through a natural generalization of these frameworks.

A particularly significant result is the definition of equilibrium, inelastic, and viscous stress tensors in Riemannian space. These have been formulated by introducing the strain tensor into spacetime and applying our non-equilibrium thermodynamic model with internal variables. From a physical point of view, we have demonstrated that the strain tensor, whether elastic or inelastic, acquires new interpretative meaning in the presence of gravitation, as its behavior in a proper system does not necessarily remain unchanged in a curved spacetime.

Based on these considerations, we were able to specify the functional dependence of entropy density in a Riemannian setting, thereby allowing for the definition of temperature within general relativity. Finally, the corresponding entropy production was derived.

These results are consistent with both the principle of general covariance and the equivalence principle. It is worth emphasizing that the results presented here can be applied to astrophysical scenarios where special relativity no longer suffices, for example, in the study of black holes.

## Data Availability

The original contributions presented in this study are included in the article. Further inquiries can be directed to the corresponding author.

## References

[1] De Groot S.R., Mazur P. (1984). Non-Equilibrium Thermodynamics.

[2] Kluitenberg G.A. (1968). A thermodynamic derivation of the stress-strain relations for burgers media and related substances. Physica.

[3] Kluitenberg G.A. (1962). Thermodynamical theory of elasticity and plasticity. Physica.

[4] Ciancio V., Farsaci F., Rogolino P. (2009). Phenomenological approach on wave propagation in dielectric media with two relaxation times. Physica B.

[5] Ciancio V., Farsaci F., Rogolino P. (2010). On a thermodynamical model for dielectric relaxation phenomena. Physica B.

[6] Rogolino P., Farsaci F. (2017). Relativistic phenomenological equations and transformation laws of relative coefficients. AAPP Atti Accad. Peloritana Pericolanti Classe Sci. Fis. Mat. Nat..

[7] Rogolino P., Farsaci F. (2016). A special relativistic approach of non equilibrium thermodynamics with internal variables. Appl. Sci..

[8] Ott H. (1963). Lorentz-Transformation der Warme und der Temperatur. Z. Phys..

[9] Ciancio V., Farsaci F. (2011). Relativistic elastic tensor BSG¤ Proceedings 18. Proceedings of the International Conference “Di®erential Geometry-Dynamical Systems 2010” (DGDS-2010).

[10] Tolman R.C. (1958). Relativity, Thermodynamics and Cosmology.

[11] Tolman R.C. (1933). On the Interpretation of Heat in Relativistic Thermodynamics. Phys. Rev..

[12] Tolman R.C. (1928). On the Extension of Thermodynamics to general relativity. Proc. Natl. Acad. Sci. USA.

[13] Finzi B., Pastori M. (1961). Calcolo Tensoriale ed Applicazioni.

[14] Kopff A. (1923). I Fondamenti Della Relatività Einsteiniana.

[15] Jorne J., Jorne P.S. (1983). Reltivistic thermodynamics of irreversible processes. Chem. Commun..

[16] Landau L.D., Lifsits E.M. (1981). Teoria dei Campi 1981.

[17] Moller C. (1952). The Theory of Relativity.

[18] Levi Civita T. (1986). Fondamenti di Meccanica Relativistica.

[19] Weinberg S. (1972). Gravitation and Cosmology.

[20] Einstein A. (2015). Il Significato Della Relativitá.

[21] Pauli W. (2008). Teoria Della Relativitá.

[22] Muschik W., Borzeszkowski H.V. (2009). Entropy identity and equilibrium conditions in relativistic thermodynamics. Gen. Relativ. Gravit..

